# Exploring the effects of spatial autocorrelation when identifying key drivers of wildlife crop-raiding

**DOI:** 10.1002/ece3.837

**Published:** 2014-02-07

**Authors:** Anna Songhurst, Tim Coulson

**Affiliations:** 1 Department of Life Sciences, Imperial College LondonSilwood Park, Ascot, Berkshire, SL5 7PY, U.K; 2 Ecoexist ProjectP. O. Box HA122HAK, Maun, Botswana; 3 Department of Zoology, University of OxfordTinbergen Building, South Parks Road, Oxford, OX1 3PS, U.K

**Keywords:** Elephant, Generalized Linear Model, Human–wildlife conflict, Okavango Delta, spatial scale

## Abstract

Few universal trends in spatial patterns of wildlife crop-raiding have been found. Variations in wildlife ecology and movements, and human spatial use have been identified as causes of this apparent unpredictability. However, varying spatial patterns of spatial autocorrelation (SA) in human–wildlife conflict (HWC) data could also contribute. We explicitly explore the effects of SA on wildlife crop-raiding data in order to facilitate the design of future HWC studies. We conducted a comparative survey of raided and nonraided fields to determine key drivers of crop-raiding. Data were subsampled at different spatial scales to select independent raiding data points. The model derived from all data was fitted to subsample data sets. Model parameters from these models were compared to determine the effect of SA. Most methods used to account for SA in data attempt to correct for the change in *P*-values; yet, by subsampling data at broader spatial scales, we identified changes in regression estimates. We consequently advocate reporting both model parameters across a range of spatial scales to help biological interpretation. Patterns of SA vary spatially in our crop-raiding data. Spatial distribution of fields should therefore be considered when choosing the spatial scale for analyses of HWC studies. Robust key drivers of elephant crop-raiding included raiding history of a field and distance of field to a main elephant pathway. Understanding spatial patterns and determining reliable socio-ecological drivers of wildlife crop-raiding is paramount for designing mitigation and land-use planning strategies to reduce HWC. Spatial patterns of HWC are complex, determined by multiple factors acting at more than one scale; therefore, studies need to be designed with an understanding of the effects of SA. Our methods are accessible to a variety of practitioners to assess the effects of SA, thereby improving the reliability of conservation management actions.

## Introduction

One of the biggest challenges in conservation today is managing situations where people and wildlife utilize the same space and compete for similar resources (Balmford et al. 2001; Sitati et al. 2005; Woodroffe 2005). As human settlements grow and protected areas become surrounded by human-dominated landscapes, human–wildlife conflict (HWC) increases and involves a growing number of wildlife species, particularly large mammals (Linnell et al. 1999; Sitati et al. 2005; Hegel et al. 2009). Large mammals generally require a large amount of space, and due to their physiology and energy requirements, also need to consume large quantities of food and water each day (Owen-Smith 1988; Sukumar 1990). It is not surprising therefore that when these animals live in areas surrounded by a burgeoning human population, they frequently compete with humans for limited resources such as space, water, and food (Hoare 2000; Conover 2002; Hegel et al. 2009). Despite an increase in the extent of HWC situations (Hoare 1999b; Madden 2004), it is still difficult to reliably predict where conflict is going to occur, or in the case of crop-raiding, for example, what makes a field susceptible to attack (Smith and Kasiki 1999; Sitati et al. 2003; Hegel et al. 2009).

Crop-raiding by wild animals occurs all over the world albeit with different species perpetrators. Studies conducted on a variety of wildlife species causing crop destruction have investigated factors and determined patterns that may help in predicting cases of HWC. Despite an array of research techniques used, common themes arising from such studies indicate that temporal and spatial patterns of wildlife crop-raiding appear to vary across space and time and may be influenced by animal density (Naughton-Treves 1998; Hegel et al. 2009), area of cultivated land (Tourenq et al. 2001), and/or the location of fields in relation to landscape features, such as water availability, protected areas, and wildlife habitat, (Lahm 1996; Naughton-Treves 1998; Smith and Kasiki 1999; Hill 2000; Saj et al. 2001; Linkie et al. 2007; Cai et al. 2008; Guerbois et al. 2012). Other factors, such as, season, guarding intensity, human density, hunting, isolation of fields, and crop type, have also been found to effect the degree of damage a field receives (Newmark et al. 1994; Naughton-Treves 1998; Hill 2000; Warren et al. 2007).

**Figure 1:**
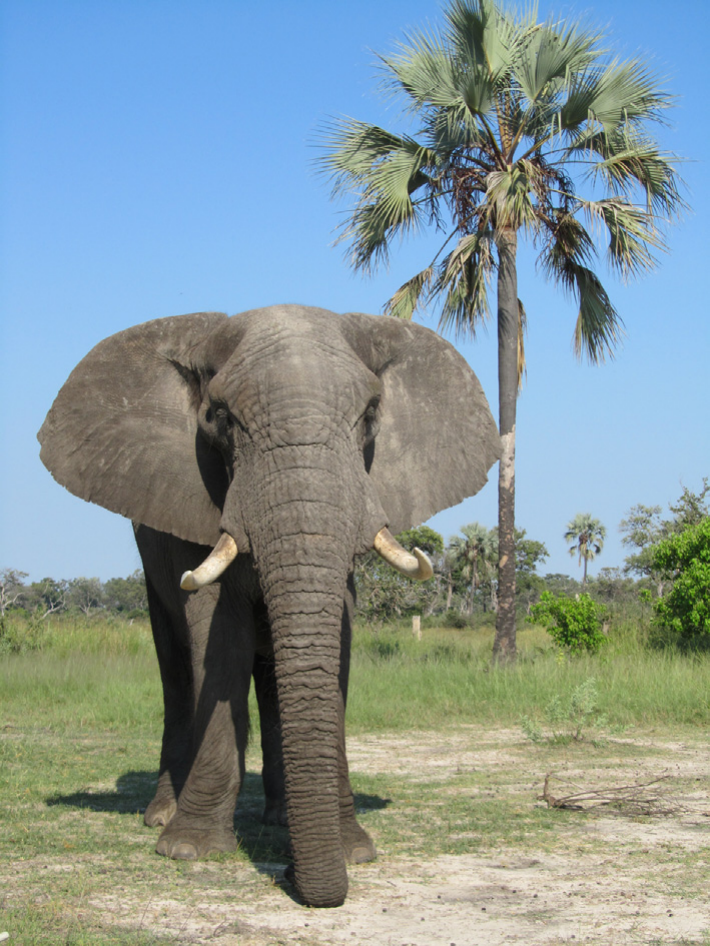
African Elephant *Loxodonta africana*

HWC is a spatial phenomenon and it is therefore important to investigate the effects of spatially explicit factors on its distribution. For example, a variety of factors have been identified in studies in east and west Africa, which may influence spatial patterns of crop-raiding by African elephants *Loxodonta africana* Blumenbach (Fig. 1) and *Loxodonta cyclotis* Matschie. In Kenya, farms that had been raided in the past, were larger and bordered by fences, were more likely to be raided by elephant and farms with greater guarding effort were less likely to be raided (Sitati et al. 2003). Both occurrence and intensity could be predicted on the basis of the area under cultivation and, for male elephant groups, proximity to major settlements (Sitati et al. 2005). A recent study also found that the occurrence of crop-raiding was predicted by settlement density, distance from daytime elephant refuges, and percentage of cultivation (Graham et al. 2010), while in Zimbabwe, distance to a protected area was the most influential determinant (Guerbois et al. 2012). Four farming variables increased the risk of a farm being raided by elephant in Ghana, distance to the national park, area of cultivation, number of crops, and degree of farm's isolation (Barnes et al. 2005). Nutritional stress, water availability, and mineral deficiency have also been identified as drivers or triggers of crop-raiding (Osborn 2004; Chiyo et al. 2005; Rode et al. 2006). It is evident from these studies that the occurrence of elephant crop-raiding appears to be influenced by the field location, farmer mitigation effort, number and types of crops planted, and the availability of water and natural forage. However, the effects of spatial autocorrelation (SA) on such spatially related drivers of crop-raiding have not yet been fully addressed in terms of its impact on the regression estimates, and therefore, how influential such drivers actually are in predicting wildlife crop-raiding.

Incidents of HWC, particularly elephant crop-raiding, are rarely randomly distributed over space. They usually show some degree of spatial clustering. For example, an elephant may raid more than one field on a particular night, and their behavior toward a field may be influenced by factors from neighboring fields (i.e., mitigation used) (Sitati et al. 2003). When such observations, drawn from different locations, are not independent from one another, they can be described as spatially dependent and SA arises (Cliff and Ord 1981). Positive SA (data collected at locations closer together are more similar) makes parametric statistical tests too liberal, and they can often produce more apparently significant results than the data actually justify (Cressie 1993; Fortin and Dale 2009). Linear estimators, correlation coefficients, and variances can also be affected by the presence of SA (Cliff and Ord 1981; Dutilleul 1993; De Knegt et al. 2010). In order to understand the spatial patterns of wildlife crop-raiding incidents, one needs to be able to separate the spatial structure of data, due to environmental factors (which are the patterns we are interested in), from that due to SA generated by the processes themselves (which can lead to spuriously significant results).

Spatial patterns of wildlife crop-raiding are therefore complex, determined by multiple factors acting at more than one scale (Hegel et al. 2009), including field locations, the ecology of crop-raiding wildlife species, and their heterogeneous use of habitats. To gain an understanding of the full extent of wildlife crop-raiding in an area, it is necessary to collect data on all raiding incidents. Determining the socio-ecological drivers of raiding from such data can, however, be problematic due to the effects of SA. Many studies aim to remove SA during analysis by choosing one field raided on a particular night in a particular location (e.g., Sitati et al. 2003, 2005) or average away SA using mixed-effects models (e.g., Guerbois et al. 2012) to correct for changes in significance levels or use spatial statistical methods to take SA into account in tests of statistical significance (for a complete review see Dormann et al. 2007); however, the actual effect of SA on such HWC data has been seldom investigated. For example, the scale used for analyses of HWC studies could affect the residual SA (De Knegt et al. 2010) and hence lead to spurious conclusions about factors influencing conflict intensity or extent.

This study aims to illustrate methods that are accessible to a variety of practitioners (scientists and wildlife managers alike) and can be used to explore the effects of SA on wildlife crop-raiding data in order to facilitate the design of future HWC studies. We collected data on attributes and position of fields, crop types grown, farmer characteristics, and mitigation measures used in an attempt to elucidate key drivers of crop-raiding by elephants in Botswana. The objectives of the study were to a) examine whether raided fields were distributed randomly in the study area, b) identify characteristics of elephant crop-raiding in the study area, c) investigate factors affecting the susceptibility of a field to crop-raiding, and d) determine the effect of SA on wildlife crop-raiding data.

## Materials and Methods

### Study area

**Figure 2:**
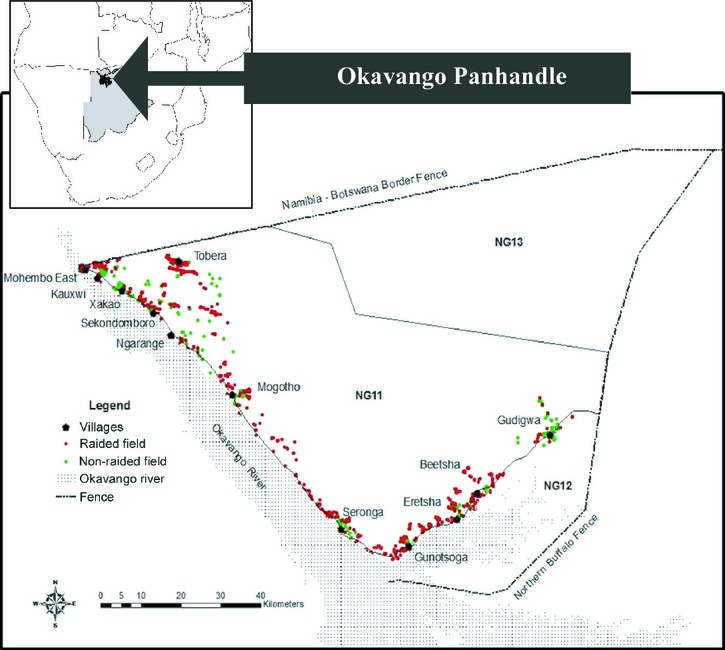
Map of the study area, with raided (red points) and nonraided (green points) field distributions in 3 years (2008–2010) illustrated.

The study was conducted on the eastern side of the Okavango Delta Panhandle (ODP), where the Okavango River reaches the Okavango Delta in Botswana, between January 2008 and July 2010 (see Fig. 2). The eastern ODP covers an area of 8732 km^2^ and consists of controlled hunting areas NG11, NG12, and NG13. The Namibian border marks the northern boundary, and the northern buffalo fence marks the southern boundary (UTM Zone 34 7910000 – 7990000 South and 580000 – 710000 East).

Rainfall averages 360-500 mm annually and generally falls between mid October and March. Mean monthly maximum temperatures range from 26.1 to 35.1°C (Department of Meteorology, 2010). Deep Kalahari sands dominate throughout the study area, and main vegetation types include *Colophospermum mopane* woodland, *Terminalia sericea* sandveld woodland, *Acacia sp*. woodland, dense *Baikiaea plurijuga,* and *Burkea africana* woodland, and Riverine woodland (e.g., *Diospyros mespiliformis; Acacia nigrescens*) (Mendelsohn and Obeid 2004). Subsistence agriculture occurs on fertile soils in lower depressions near the Okavango River and in-land <14 km from the main road.

The 2001 census recorded 15,718 people living in the eastern ODP (CSO 2001). There are 12 main villages (population >500 people) in the area, extending from Mohembo-East to Gudigwa, including Kauxwi, Xakao, Tobera, Sekondomboro, Ngarange, Mogotho, Seronga, Gunotsoga, Eretsha, and Beetsha, with additional settlements occurring between villages. Depending on annual rainfall, the planting of crops occurs between November and January and harvesting occurs between April and June. Elephants range throughout the eastern ODP. The population was estimated to be 15,429 (± 2008 SE) elephants in 2010 with a density of 1.77 elephants^−1^ km (Songhurst and Chase 2010). Elephants use distinctive pathways to get to the Okavango River (Loarie et al. 2009; Songhurst 2012), enabling us to investigate the role of elephant movement as a factor in determining spatial patterns of human–elephant conflict (HEC) in this study area.

### Independent primary HEC data

In order to establish a reliable independent conflict reporting system for this study, we used the standardized data collection protocol of the International Union for the Conservation of Nature (IUCN) (Hoare 1999a) to collect primary data on HEC. All fields raided by elephant between January 2008 and May 2010 were visited by both the local enumerator and the principal investigator (A.S) to ensure consistency and reliability of data collection. Details on each damage incident were recorded on standardized data collection forms. The average pace size of each enumerator was measured, and the area of all fields and damaged portions of the fields were estimated in square meters using enumerator paces (Hoare 1999a). Each damage incident was geo-referenced in Universal Transverse Mercator (UTM) coordinates using a Garmin high-sensitivity global positioning unit (Garmin Corp., Ulathe, KA) and location details recorded.

### Spatial distribution of fields

Raided and nonraided fields from the whole study period were plotted to examine the spatial distribution of fields. The distribution of nearest neighbor distances was calculated as a first step in determining whether there is any evidence to reject the null hypothesis of complete spatial randomness of raided fields. The observed number of raided fields was then distributed randomly across the mapped fields, and the mean nearest neighbor distance (MNND) from this random sample was calculated. This was repeated 1000 times, and the frequency distribution of MNND plotted. This enabled us to see where in this distribution, the observed mean distance was and determine whether fields were raided at random given the distribution of fields in the study area. If fields are not raided at random within the distribution of raided fields, then it is evident that the actual locations (coordinates) of observational data matter in explaining the spatial arrangement. The spatial distance was plotted against temporal distance between raided fields, to help identify the spatial scale at which to define a raid location. Moran's *I* statistic was calculated to test whether nearby observations tended to have similar attributes or to be more clustered than expected from randomness alone and significance tested with a *Z*-test.

### Susceptibility to elephant crop-raiding

A comparative survey of raided and nonraided fields was carried out to determine factors affecting the susceptibility of a field to being raided by elephant. All fields reporting elephant raids were visited over three crop seasons. At the end of each crop season, a selection of nonraided fields in different localities was identified by enumerators in each village. Although this was not strictly random, it is comparable to methods used in other studies (Tourenq et al. 2001; Barnes et al. 2005; Chiyo et al. 2005; Sitati et al. 2005) and was logistically feasible.

Generalized linear models (GLMs), with binomial errors, were used to explore a range of factors affecting the susceptibility of fields to elephant crop-raiding. Initially, univariate analyses were carried out with all explanatory variables. Significant variables (*P *<* *0.05) were then used in multivariate analysis to investigate correlates explaining the susceptibility of a field to crop-raiding. The maximum model for two-way interactions was fitted and simplified by stepwise deletion of nonsignificant terms (Crawley 2007). The elephant raid (successful or not) on a particular field was used as the independent unit of analysis in statistical analyses. Fields were coded as raided (1) or nonraided (0), as were mitigation methods (1 = used, 0 = not used), crop types (1 = present, 0 = not present), and other animals raiding (1 = raiding, 0 = not raiding). The data were screened for collinearity and outliers prior to analysis (using box plots and scatter plots). Influential data points were left out one at a time, and the model was refitted to check whether parameter estimates or standard errors were substantially affected. Covariates recorded were as follows: distance from field to i) permanent (river) and semipermanent (waterhole) water, (DRV and DWH, respectively), ii) next field (DNF), iii) main village (DV), iv) main road (DMR), and v) main elephant path (DEP); mitigation techniques used; number of watch huts (NWH) and guards (NG); crop types grown; field characteristics (age (FAGE), area (AREA), elevation (ELEV), number of years previously raided (NYR)); farmer details (age (AG), ethnicity (ETH), gender (G), family size (NIF), and livelihood); and predominant vegetation type around field.

### Effect of spatial autocorrelation

Initially, all data were used in the analyses. This, however, assumes no SA is present in the data. One method of removing spatial dependency among observations can be to remove samples until spatial independence has been attained (Cliff and Ord 1981). The total dataset was therefore subsampled to select one field raided on a particular night in a particular location (grid cell) at different spatial scales. Grids of varying sizes (0.5 km^2^, 1 km^2^, 1.5 km^2^, 2 km^2^, 2.5 km^2^, 5 km^2^), which would incorporate daily movement distances of elephants in the ODP [3–6 km^−1 ^day (Loarie et al. 2009)], were superimposed over the study area, and one field per grid cell chosen for the subsample (representing one field per location). This subsampling process was replicated 500 times at each spatial scale. The minimum adequate model (MAM) derived from all data was then fitted to the subsampled data. Model estimates and *P*-values from the MAM using all data were compared with estimates and *P*-values derived from subsampled data at different spatial scales to determine the effect of SA on model parameters.

### Data analysis

All data analysis was carried out using R 2.11.1 (R Development Core Team. 2010). Bootstrapping and simulation techniques were used to investigate whether a field was raided at random within the study area. GLM model fit was checked using chi-squared goodness-of-fit test, and significance determined for all analyses at *P *<* *0.05. Using *P *<* *0.05, however, could result in only a tiny percentage of deviance being explained. A second set of analysis was conducted to examine factors which explain >1% of the variance in the model, to establish biologically significant variables and interactions, which could be used as practical predictors of conflict in management actions.

## Results

### Spatial distribution of fields

**Figure 3:**
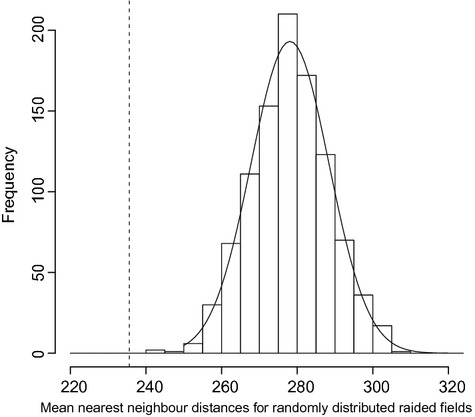
Histogram of 1000 bootstrapped mean nearest neighbor distances of simulated randomly distributed elephant raided fields.

Field (raided and nonraided) distribution exhibits a clustered pattern in the study area (see Fig. 2). The distribution of MNND of the 1000 bootstrapped samples of simulated randomly distributed raided fields is illustrated in a histogram (see Fig. 3). The MNND of observed raided fields (μ = 235.59 m) lies to the left of the distribution of randomly distributed fields outside of the 95% percentiles of the distribution (2.75% = 257.62 and 97.5% = 299.67), suggesting that raiding is spatially nonrandom and therefore location of fields influence raiding patterns.

There was a correlation (*r*_p_ = 0.067, *P *<* *0.001) between the spatial distance between raided fields and the temporal distance between raided fields. This spatio-temporal correlation generates SA in the data. The average length of time between raids for fields <0.5 km apart was 16 days, while for raided fields >2 km apart, it was 32 days.

The spatial distribution of fields within the study area is clustered, and it is, therefore, difficult to investigate the influence of SA using geostatistical measures. Moran's *I* showed significant positive SA in all fields and raided fields data (*I *=* *0.328, *P *<* *0.001 and *I *=* *0.240, *P *<* *0.001, respectively). However, for raided field data subsampled at the 0.5 km^2^, 1 km^2^, 1.5 km^2^, 2 km^2^ spatial scales, Moran's *I* decreased and then leveled out, illustrating that SA is reduced from data when subsampled at larger spatial scales.

### Characteristics of elephant crop-raiding

Over 3 years, 1421 fields were assessed; 788 raided, and 633 randomly selected nonraided fields. A total of 162.12 ha of crop were recorded as damaged throughout the 3 years with a mean amount of damage per incident of 0.23 ha (± 0.72 ha). The median proportion of damage per field was 2.02%, with a quarter of raided farms suffering less than 0.4% and a quarter suffering more than 10.1% damage.

### Key drivers of elephant crop-raiding

In multivariate analysis, the MAM containing variables explaining <1% of the overall variance (see Table 1a), retained variables DV, NYR, and pumpkin growing as having significant positive effects (fields are more likely to be raided, if they are far from the village, have been raided considerably in the past, and have pumpkins growing). It retained variables DWH, DEP, FAGE, and the presence of livestock raiders as having significant negative effects (fields less likely to be raided are older, far from waterholes, far from elephant paths, and where livestock also raid). The model shows that the raiding of a field depends on a two-way interaction between the age of a field and whether pumpkins are growing (χ^2^ = 7.47, *P *=* *0.006) as having a negative effect (older fields growing pumpkin were less likely to be raided). Two influential data points (398 and 409) were removed after the MAM was checked using residual plots.

**Table 1:** (a) The minimum adequate model of the generalized linear model containing variables explaining <1% of the overall variance for whether a field is raided or not, with binomial error structure and significance at *P *<* *0.01 and (b) estimates and *P*-values for GLM using subsampled data at different spatial scales (significant results highlighted in bold).

Variable	(a)						(b)							
	All Data						0.5 km^2^		1 km^2^		1.5 km^2^		2 km^2^	
	Estimate	SE	Z	*P*	χ^2^ Deviance	χ^2^ *P*	Estimate*	P*	Estimate*	P*	Estimate*	P*	Estimate*	P*
Intercept	0.5	0.4	1.4	0.17	1.3	0.06	1.9	0.04	1.6	0.2	2.1	0.1		
Dist. Village	**0.019**	**0.003**	**6.0**	**1.8e**^**−9**^	**38.9**	**4.3e**^**−10**^	**0.014**	**0.007**	0.012	0.05	0.009	0.3	0.011	0.2
Dist. Waterhole	**−0.02**	**0.007**	**−2.9**	**0.003**	**8.9**	**0.003**	−0.018	0.09	−0.023	0.1	−0.017	0.3	−0.027	0.2
Dist. Ele Path	**−0.039**	**0.006**	**−6.9**	**2.6e**^**−12**^	**51.9**	**5.9e**^**−13**^	**−0.043**	**2.9e**^**−6**^	**−0.042**	**5.0e**^**−4**^	**−0.043**	**2.6e**^**−3**^	**−0.036**	**3.5e**^**−2**^
Field Age	**−0.49**	**0.09**	**4.2**	**2.4e**^**−7**^	**5.1**	**2.2e**^**−16**^	**−0.642**	**0.0004**	**−0.814**	**0.001**	−0.79	0.02	−0.962	0.02
No. Years Raided	**1.221**	**0.09**	**−5.2**	**<2e**^**−16**^	**9.6**	**2.2e**^**−16**^	**1.29**	**3.8e**^**−18**^	**1.324**	**5.6e**^**−12**^	**1.489**	**2.7e**^**−8**^	**1.518**	**3.1e**^**−7**^
Pumpkin	**1.462**	**0.4**	**3.9**	**7.8e**^**−5**^	**12.7**	**0.0004**	1.134	0.09	0.689	0.4	1.281	0.2	1.062	0.4
Livestock	**−0.892**	**0.2**	**−5.8**	**6.2e**^**−9**^	**4.4**	**4.5e**^**−9**^	−0.687	0.01	−0.814	0.03	−0.896	0.05	−1.218	0.03
Field Age:Pumpkin	**−0.338**	**0.1**	**−2.7**	**0.006**	**7.4**	**0.006**	−0.251	0.3	−0.033	0.5	−0.228	0.4	−0.052	0.5

* Mean.

Further chi-square analysis showed that biologically significant variables and interactions (explaining >1% of overall variance), which could be used as practical predictors of conflict in management actions, were those which had a chi-square test residual deviance of >11.2. Variables retained in this analysis included DEP (χ^2^ = 51.87, *P *=* *5.92e^−13^) explaining 4.6% of variance, DV (χ^2^ = 38.98, *P *=* *4.29e^−10^) explaining 3.5% of variance, and growing pumpkins (χ^2^ = 15.77, *P *=* *7.15e^−5^) explaining 1.1% of variance.

### Exploring the effect of spatial autocorrelation

**Figure 4:**
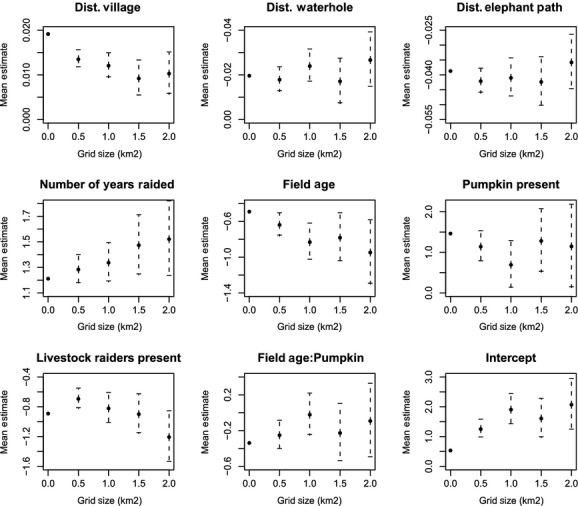
Relationships between mean parameter estimate from generalized linear model and grid size for all explanatory variables of elephant crop-raiding and the intercept.

**Figure 5:**
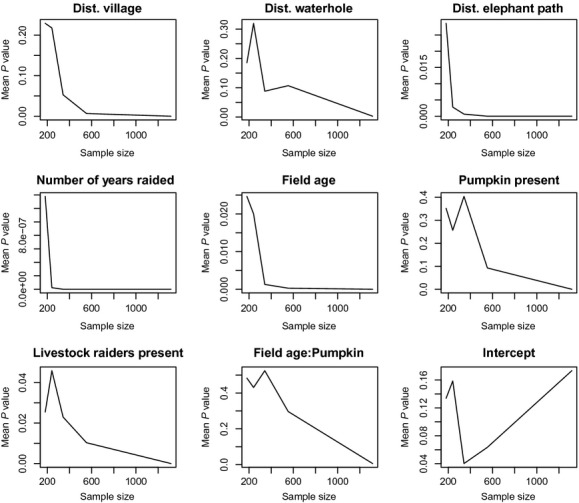
Relationships between mean *P*-value and sample size for all covariates explaining the susceptibility of a field to elephant raiding.

When subsampling data at different spatial scales to reduce the SA in the data, coarser grid sizes ≥2 km^2^ had too few data points for some variables and could not be used in subsequent analysis. Results from four grid sizes (≤2 km^2^) only are shown in Table 1b and Figures 4 and 5.

As expected, as the spatial scale for subsampling increases, the sample size of variables decreases and the standard errors around mean estimates increase for all variables. It is also evident that SA affects the size of the model estimates of different variables, with estimate values either increasing or decreasing as SA is reduced through subsampling at different grid sizes. SA consequently not only influences standard errors and *P*-values, but it also influences our biological interpretation. However, patterns of change in the size of estimates across spatial scales were not consistent across variables (see Fig. 4). The intercept increased with increasing spatial scale, indicating that the average amount of raiding appears to increase as spatial scale of subsampling increases (see Fig. 4i).

For all variables, the *P*-value decreased as sample size increased (spatial scale decreased), as expected (see Fig. 5). It is evident from this graph that even at a smaller sample size (larger spatial scale), the significance (*P*-value) of variables such as DEP, NYR, FAGE and presence of livestock do not vary to a large extent and are still statistically significant at *P *<* *0.05. There does not appear to be a strong association between patterns for the estimates and patterns for the *P*-values. The plots of mean intercept vs. grid size show that the intercept increases gradually with increasing grid size.

**Figure 6:**
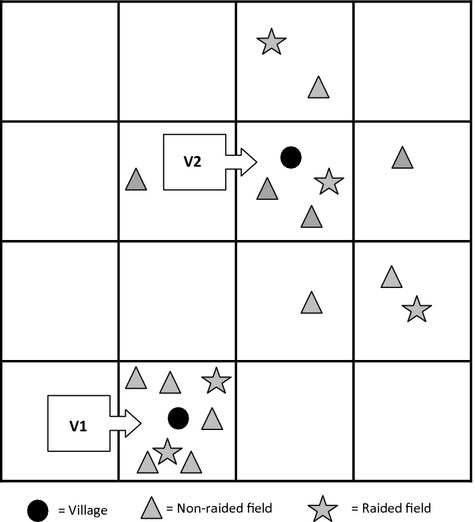
Schematic diagram explaining change in model estimates, and *P*-values of distance to village covariate when subsampling to reduce effect of spatial autocorrelation.

These differences in model estimates (and *P*-values) of variables are driven by the spatial distribution of fields in an area. For example, if fields are close to one village (V1 in Fig. 6) yet spread out around another village (V2 in Fig. 6), when subsampling one raided/nonraided field per grid cell, the sample size will decrease dramatically for V1 (from *n* = 5 to *n* = 1 nonraided and *n* = 2 to *n* = 1 raided) but remains relatively stable for V2 (from *n* = 7 to *n* = 6 nonraided and *n* = 3 for raided). The model estimates for the variable distance to village will therefore be dramatically affected by such subsampling. When all data are used in analyses, there will be a lot more variance in the data, but when we throw data points away through subsampling, the intercept and slopes of model variables change. Biologically, when we subsample data, so they conform to statistical assumptions, we potentially lose valuable information. We consequently advocate reporting both regression estimates and *P*-values across a range of spatial scales to help biological interpretation.

**Figure 7:**
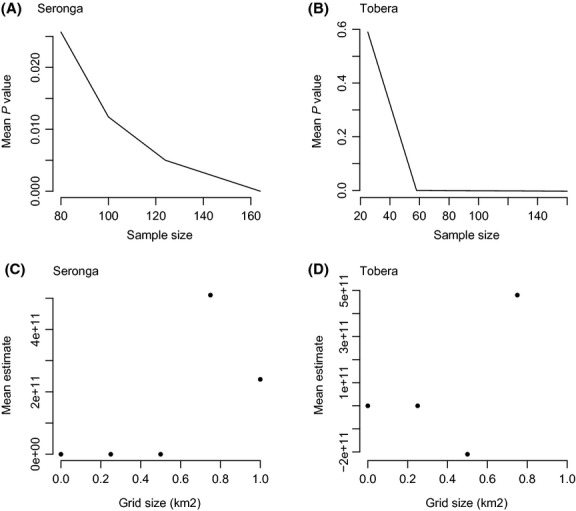
Relationships between: i) mean *P*-value and sample size for distance to village covariate for two villages with different distributions of fields (A) Seronga (spread out) and (B) Tobera (clumped); and ii) mean parameter estimate from generalized linear model and grid size for distance to village variable in the same two villages (C) Seronga and (D) Tobera.

If we use the distance to village variable and investigate what happens to model estimates and *P*-values where fields are dispersed around the village (e.g., Seronga) and compare this to a village where fields are more clumped (e.g., Tobera), it is evident that *P*-values do indeed decrease more gradually for areas with dispersed fields compared to clumped (see Fig. 7A,B), and the model estimates change at a lower grid size for Tobera than Seronga (see Fig. 7C,D).

## Discussion

Our study indicates that there is spatial variation in the SA in crop-raiding data, and therefore, the spatial distribution of fields should be considered when choosing the spatial extent of analyses for wildlife crop-raiding studies. Different spatial distributions of fields and explanatory variables are affected in different ways through subsampling to reduce SA in the data. Consequently, it would be advantageous to assess HWC incidents at a village level where clumped field distributions could be separated from dispersed distributions. Such localized research would facilitate the development of relevant crop vulnerability models and enable effective management strategies to be designed at a local level. It is advocated that all data be used to find the MAM containing significant correlates of raiding and encourage the use of subsampling data at different spatial scales to reduce the effect of SA, thereby avoiding the need to define an arbitrary location of a raid. However, because SA affects both significance levels and model estimates, it is advised that both values be reported across a range of spatial scales for all statistically significant explanatory variables to aid in the biological interpretation of the data.

### Effect of spatial autocorrelation in wildlife crop-raiding data

We found that not only the *P*-values in regression analysis of elephant crop-raiding data were affected by SA, but also the regression estimates changed when data were subsampled at coarser spatial scales (when SA was reduced). This highlights the importance of using an appropriate spatial scale in the analyses of such data, as scale mismatches can affect our understanding of spatial patterning in ecological studies (De Knegt et al. 2010). Legendre et al. (2002) found that if SA is present in both the response and explanatory variables in regression analysis, then the significance of correlation and regression coefficients can be disturbed. We know that SA was present in the response variable, but results from Legendre et al. (2002) would suggest that SA was present in the explanatory variables as well. When choosing one raided/nonraided field per cell, the degrees of freedom are reduced, and therefore, the significance levels increase, but we also found that the regression estimates changed. The regression estimates change if one excludes most of the data with, for example, a 1 (raided) rather than a 0 (not raided) by sampling only one point from one cell. There is a lot more variance in the response variable (raided/not raided) when one uses all the data, but when data points are discarded during subsampling, the variance decreases, meaning that the intercept changes and also the slope (regression estimate). Many methods used to account for SA in data attempt to correct for the change in significance levels, yet our study shows that by subsampling data at broader spatial scales, we can identify changes in regression estimates as well, which is important for the biological interpretation of wildlife crop-raiding data.

The average intercept appeared to increase at broader spatial scales of subsampling. The intercept effectively gives an estimate of the amount of raiding occurring, and therefore, this result infers that there are larger amounts of raiding occurring at broader spatial scales. It is important to note that the amount of raiding occurring may be inflated when subsampling in this way. It is also evident that patterns in the changes in significance levels and regression estimates vary for different explanatory variables depending on the spatial distribution of fields and explanatory variables. Our results suggest that there is spatial variation in the pattern of SA in crop-raiding data. For areas where the fields are spatially dispersed, there is a less dramatic effect on both *P*-values and regression estimates when SA is reduced, than for areas where fields are clumped. Extra caution is therefore needed when subsampling data from fields with a clumped distribution. Such findings indicate that models should be developed for spatial areas that contain fields displaying similar (i.e., all clumped) spatial distributions rather than varied (i.e., dispersed and clumped), for example, at a village scale rather than on a broader (ward/region/countrywide) scale where field distribution is likely to vary among villages.

Previous studies investigating spatial correlates of crop-raiding have used districts or known elephant ranges to define the spatial extent to be used in the analysis (Hoare 1999b; Smith and Kasiki 1999; Sitati et al. 2003; Graham et al. 2010), and it has been found that it is easier to identify predictors of HEC at broader spatial extents (Sitati et al. 2003; Graham et al. 2010). Graham et al. (2010) suggested that if resources are limited, then the use of such parameters to define spatial extent in the analysis of HEC data is adequate for identifying broad priorities for management intervention. Our results illustrate, however, that caution should be exercised when defining spatial extents for analysis based on the above criteria, because the effect of SA in the data varies depending on the spatial distribution of fields, and therefore, field distribution in an area should also be considered when choosing the spatial extent for analysis.

### What is a “location” of a raid?

One method to remove SA and avoid pseudoreplication is to take a subsample of data (Hurlbert 1990; Hoare 1999b). In the case of crop-raiding data, a suitable subsample would be to select one field raided on a particular night in a particular location (Sitati et al. 2005). However, it is difficult to determine what a “location” of an independent raid is. For example, from an elephant's perspective, the location of a raid on a particular night could incorporate fields within the nights foraging expedition or movement route to available water. In the ODP, elephants can move up to 40 km/day roundtrip, with average daily movements varying in the dry (6 km/day) and wet seasons (3 km/day) (Loarie et al. 2009). A location of one elephant foray on a particular night could therefore be considered to include all raided fields within a 3–40 km^2^ radius. Yet, it is also likely that more than one elephant herd are responsible for crop-raiding incidents on any particular night, and therefore, even fields within a 500 m^2^ radius could be considered independent raids because they were conducted by different groups of elephants.

For management purposes, a “location” or sampling unit could be considered to be an individual field as used by Graham et al. (2010). We therefore included data from all elephant raiding incidents in initial analyses to derive correlates of crop-raiding at the field level. Using all data, however, assumes no SA is present in the data, and therefore, it is advised that subsampling at different spatial scales be used when using GLMs to analyze HWC data to reduce the effects of SA without having to define an arbitrary “location” of a raid.

### Robust key drivers of elephant crop-raiding

In the ODP, elephant crop-raiding was spatially nonrandom indicating that the location of fields influenced the pattern of raiding and SA was present in our raided field data. We found that not only the presence of an elephant pathway, as previously found by Guerbois et al. (2012), but also the actual distance of a field to an elephant pathway influenced elephant crop-raiding patterns. Fields further from elephant pathways were less likely to be raided by elephants, and fields that had been raided frequently in the past were more likely to be raided again. Elephants have long memories (McComb et al. 2001) and often utilize traditional movement routes to get to watering or foraging sites. One explanation for both these results could therefore be that elephants are returning to fields they remember having raided successfully in the past, which were either close to these traditional movement paths, or movement paths have been altered to travel close to these foraging sites. At finer spatial scales, the age of a field had been raided in the past also affected the likelihood of a field being raided by elephant during the study period, which could also indicate that elephants are remembering older fields raided in previous years. Similar patterns, where fields raided in the past were more likely to be raided, have also been identified in Kenya (Sitati et al. 2005). Naturally, there is an increased likelihood of elephants using elephant pathways to encounter fields closer to these pathways than fields further away. Therefore, it could also be opportunistic foraging behavior (e.g., Osborn (1998), which explains why fields closer to elephant pathways are more likely to be raided.

### Recommendations for management

Understanding spatial patterns of crop damage is paramount for designing better mitigation and land-use planning strategies. Our study revealed that varying patterns in spatial autocorrelation drive the varying relationships between crop-raiding and independent explanatory variables that Graham et al. (2010) highlighted. We agree with Guerbois et al. (2012) and Graham et al. (2010) that future spatial analysis of HEC should be conducted at varying spatial extents to reveal broad and local level patterns, however, it is important to consider the spatial distribution of fields when choosing such extents due to the variation of SA in crop-raiding data. To identify robust key drivers of elephant crop-raiding, incidents should be assessed where clumped and dispersed field distributions can be separated for analyses, and therefore, a village level assessment would be advantageous. If SA is not taken into account during both the planning and analyses stage of an HWC study, explanatory variables may appear more influential in driving crop-raiding incidents than they really are. Such spurious conclusions could lead to farmer mitigation efforts being concentrated in less affected areas or misinformed management recommendations being given to wildlife management and land authorities, which will result in a waste of limited resources and farmer effort.

We identified key drivers of elephant crop-raiding in the ODP to be distance from elephant pathways and history of raiding, which will facilitate both the short-and long-term management of HEC in this area. Mitigation efforts can target higher risk fields close to pathways and those raided frequently in the past, in order to focus time and resources. In addition, elephant pathways that are frequently used should be allocated a free movement buffer zone along their route, where arable land allocation should be prohibited, thereby reducing the likelihood of fields being allocated closer to main elephant pathways, and subsequently, their likelihood of being raided. Such land-use interventions will certainly reduce field vulnerability to crop-raiding and ensure free elephant movement along critical routes in agricultural landscapes.
